# *VSG* mRNA levels are regulated by the production of functional VSG protein

**DOI:** 10.1016/j.molbiopara.2020.111348

**Published:** 2021-01

**Authors:** Isabella E. Maudlin, Steve Kelly, Angela Schwede, Mark Carrington

**Affiliations:** a Department of Biochemistry, University of Cambridge, Tennis Court Road, Cambridge CB2 1QW, United Kingdom; b Department of Plant Sciences, University of Oxford, South Parks Road, Oxford, OX1 3RB, United Kingdom

**Keywords:** *Trypanosoma brucei*, VSG mRNA

## Abstract

The bloodstream form of *Trypanosoma brucei* persists in mammalian hosts through a population survival strategy depending on antigenic variation of a cell surface coat composed of the variant surface glycoprotein (VSG). The integrity of the VSG coat is essential and blocking its synthesis results in a cell division cycle arrest just prior to cytokinesis. This observation indicates that VSG levels are monitored and that the cell has mechanisms to respond to a disruption of synthesis. Here, the regulation of *VSG* mRNA levels has been investigated by first measuring *VSG* mRNA copy number, and second using ectopic expression of *VSG* transgenes containing premature termination codons. The findings are that (i) *VSG* mRNA copy number varies with the identity of the VSG and (ii) a pathway detects synthesis of non-functional VSG protein and results in an increase in *VSG* mRNA levels.

## Highlights

• *VSG* mRNA copy number varies with the identity of the VSG.
• Early premature termination codons result in degradation of *VSG* mRNA.
• Late premature termination codons translated to produce incomplete VSG result in an increase in *VSG* mRNA.
• There is a feedback pathway between non-functional VSG production and *VSG* mRNA levels.

## 1 Introduction

*Trypanosoma brucei* can sustain long-term infections in mammalian hosts by antigenic variation of the variant surface glycoprotein (VSG) that forms a coat covering the entire surface of the cell [1]. The active *VSG* gene is located near a telomere at the 3′ end of a transcription unit known as a bloodstream expression site (BES). There are 10–20 BESs in *T. brucei* genomes but only one BES is transcribed at any one time [2,3]. Antigenic variation occurs when a low frequency event, either gene conversion in the active BES or a less well characterised epigenetic switch to an alternative BES, results in transcription of a different *VSG* gene [4]. The processes involved in antigenic variation and monoallelic VSG expression have been studied in some detail, the active BES is transcribed by RNA polymerase I (RNAPI) [5] and is located in the nuclear expression site body (ESB), a compartment containing RNAPI lying outside the nucleolus [6]. Expression of protein coding genes by RNAPI evolved in a cell in which the maturation of every cytoplasmic mRNA included *trans*-splicing of a capped 39 nucleotide exon to the 5′ end [7], thus the first 39 bases of a *VSG* mRNA are transcribed by RNAPII and the remainder by RNAPI. Presumably, RNAPI transcription allows the exceptionally high levels of *VSG* mRNA in the cell to be transcribed from a single gene. The precise details of VSG monoallelic expression, both the default silencing and the escape from silencing by the active BES, are less well understood but involve a VEX/CAF complex identified in a whole genome RNAi screen for loss of monoallelic VSG expression [3,8].

There is strong evidence that mechanisms have evolved to ensure that the supply of newly synthesised VSG is adequate to maintain the densely packed coat as new plasma membrane is added during cellular growth. Blocking VSG synthesis by RNAi knockdown of the mRNA or by blocking translation using a morpholino oligonucleotide results in cell division cycle arrest at the onset of cytokinesis [9,10]. It is assumed that cytokinesis is the point in the cell cycle where there is the highest demand for new VSG coated membrane. Trypanosomes with a common genetic background but expressing different VSGs proliferate at different rates in identical culture conditions, providing evidence that the identity of the expressed VSG can affect cellular growth rates with some VSGs effectively constraining proliferation [11]. As VSGs constitute 10–20 % of total protein and VSGs have very different amino acid sequences, the rate of successful folding and export from the endoplasmic reticulum may be limiting, thus affecting growth rates. Studies have reached mixed conclusions, either that VSG protein is synthesised in amounts greater than is needed and the excess degraded [12,13] whereas another report found that just sufficient is synthesised [14].

As might be expected for an mRNA encoding a highly abundant protein, *VSG* mRNA has a long half-life in proliferating cells with measurements ranging from 4.5 h [15] down to 1 h [16]. Its levels can be modulated by the cell as expression of a second *VSG* from a transgene reduces steady state mRNA levels from the endogenous *VSG* gene. Inducible expression of a T7 RNA polymerase-driven *VSG6* (also known as VSG121) transgene located in the non-transcribed spacer of a ribosomal RNA gene cluster was shown to cause a rapid decrease in endogenous *VSG2* (also known as VSG221) mRNA levels and a slower transcriptional attenuation of the active BES dependent on the activity of DOT1B (disruptor of telomeric silencing-1B) [17]. It is not clear whether attenuation of the active VSG expression site is an entirely intranuclear event or if there is a cytoplasmic contribution. In a second set of experiments, insertion of a *VSG117* gene in various locations in the genome of cells expressing VSG2 resulted in a reduction of *VSG2* mRNA proportional to the amount of *VSG117* mRNA expressed [16]. These observations imply that the total VSG mRNA level is regulated and that the mechanism can recognise different *VSG* mRNAs despite their diverse sequences. The only obvious conserved motif in *VSG* mRNAs is a 16 nucleotide sequence (16-mer) in the 3′UTR close to the poly(A) tail. Although the 16-mer is necessary for the very high levels of cytoplasmic *VSG* mRNA [16], no mechanism has been characterised for how it is involved in positive regulation of high levels of *VSG* mRNA and it is unknown whether it is also involved in down regulation when a second *VSG* mRNA is expressed.

Here, expression of a second *VSG* from transgenes containing a premature termination codon (PTC) at various locations in the open reading frame was used to produce cytoplasmic degradation of *VSG* mRNA [18,19] and the consequent effect on *VSG* mRNA measured. In yeast and metazoa, recognition of a PTC during translation triggers a reduction in the half-life of an mRNA via the nonsense-mediated decay (NMD) pathway mediated by a complex containing the RNA helicase UPF1 (reviewed in [20,21]). In yeast, NMD is not a binary process and only rarely is a PTC-containing mRNA completely absent from a cell (for example [22]). The degree of reduction in the PTC-containing mRNAs is affected by the location of the PTC within the open reading frame, the nearer to the initiation codon the more probable that NMD will be triggered on any round of translation [23]. In trypanosomes, investigation of PTC-containing mRNAs showed a similar relationship between PTC location in an ORF and reduction in mRNA levels but any role for the UPF1 homologue remained ambiguous [24].

Expression of PTC-containing *VSG* transgenes and measurement of transgene and endogenous *VSG* mRNA levels confirmed the presence of a pathway that reduced PTC-containing *VSG* mRNAs, with the decrease being proportional to the proximity of the PTC to the initiation codon. Measurements of the *VSG* mRNA in wild type cells showed that VSG6 expressing cells have a higher copy number than VSG2 expressers and that double expressers had intermediate levels. An unexpected finding was that expression of *VSG* transgenes with PTCs close to the C-terminus resulted in increases in both transgenic and endogenous *VSG* mRNAs. Together, these experiments provide further evidence for a pathway that decreases PTC-containing mRNAs, and a second pathway regulating *VSG* mRNA levels that is linked to production of functional VSG protein.

## 2 Materials and methods

### 2.1 Plasmids

The sequence of the VSG6 BES was taken from Genbank entry FM162569 and VSG2 BES from FM162566. Genomic DNAs from yeast containing these cloned telomeres (a kind gift of Gloria Rudenko) were used as templates for PCR reactions to recover the fragments described below. Constructs to introduce a *VSG2* transgene were based on the plasmid p3952. Digestion of p3952 with restriction enzymes Acc65I and SacI released a 4.6 kbp fragment (Supp. Fig. 1A) that contained, in order: bases −800 to −300 upstream of the VSG6 initiation codon (5′ targeting); XhoI site; bases -182 to -1 upstream of the VSG2 initiation codon (VSG2 splice acceptor and 5′UTR); an SphI site; VSG2 ORF; PacI site; bases 1–615 downstream of the VSG2 termination codon (VSG2 3′UTR and polyadenylation); XmaI site; a tubulin/blasticidin resistance cassette (alpha to beta tubulin inter-ORF, blasticidin-S-deaminase ORF, beta to alpha tubulin inter-ORF); SpeI site; bases −300 upstream to +210 downstream of the VSG6 initiation codon (3′ targeting) (see Supp. Fig. 1B for sequence). The plasmid was constructed by joining fragments using the restriction enzyme sites listed above. Subsequent changes to the VSG2 ORF used SphI and PacI. NEB Phusion DNA polymerase was used for site-directed mutagenesis according to the manufacturer’s instructions, oligonucleotides are listed in Supp. Table 1.

### 2.2 Cell culture and sample preparation

*Trypanosoma brucei* Lister 427 bloodstream form trypanosomes were used as parental cell lines throughout this study. HMI-11 [25] was used to culture bloodstream form trypanosomes with 5 μg/ml blasticidin to maintain genetically modified cell lines. All experiments were performed using logarithmically growing cells at a density of less than 1 × 10^6^ cells/ml. Preparation of whole cell lysates, western blotting, preparation of total RNA and northern blotting all used standard techniques [26] with the following details. The primary antibodies used for Western blotting were: (i) Rabbit anti-VSG2 N-terminal peptide [27] (a kind gift of Peter Overath), (ii) mouse monoclonal anti-VSG6 (a kind gift of Miguel Navarro), and (iii) mouse monoclonal L13D6 anti-paraflagellar rod proteins (PFR) 1 and 2 (a kind gift from Keith Gull). Secondary antibodies were: (i) goat anti‐Rabbit AlexaFluor 680, and (ii) goat anti‐Mouse IRDye800. The LI-COR Odyssey infrared imaging system was used to visualise western blots. For northern blots, the relevant whole VSG ORF was used as a probe. Phosphorimaging was used to quantitate RNA from northern blots, background from the blot was subtracted from the band-of-interest, and then normalised against the rRNA loading control signal.

### 2.3 RNAseq for transcript abundances

Total RNA was prepared as above for single sample RNAseq to estimate *VSG* mRNA abundance. The cDNA libraries were prepared and sequenced at the Beijing Genomics Institute (Shenzhen, China) [28] In brief, polyadenylated RNA was purified from total RNA, converted to cDNA using random hexamer primers sheared and size selected for fragments ∼200 bp in length using the Illumina TruSeq RNA Sample Preparation Kit v2. RNAseq of the resulting libraries was used for the determination of transcript abundances. Sequencing was performed on an Illumina Hiseq 2000 (Illumina, CA) platform. Paired end reads were subject to quality trimming and adaptor filtering using Trimmomatic [29] using the settings “LEADING:10 TRAILING:10 SLIDINGWINDOW:5:15 MINLEN:50”. The quality filtered paired-end reads were then mapped to the complete set of CDS from version 6 of *T. brucei* genome annotation using bowtie2 [30] and transcript abundances were estimated using eXpress [31]. The sequence reads are in EBI ArrayExpress accession E-MTAB-9122.

## 3 Results

### 3.1 *VSG2* transgenes were expressed and PTCs were effective in terminating translation

**Fig. 1:**
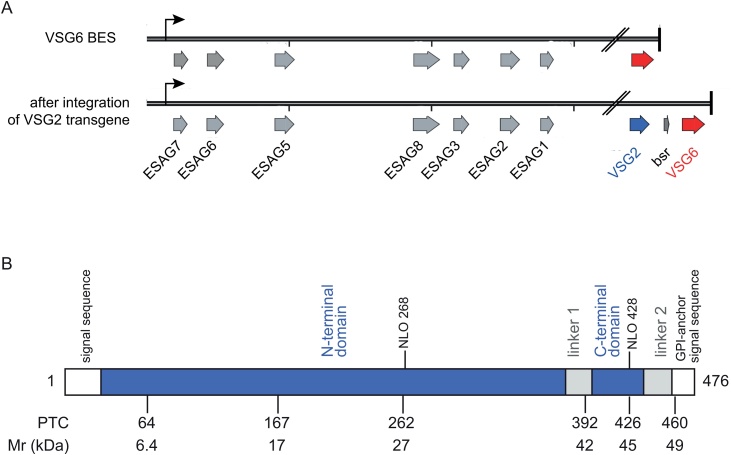
Integration of the *VSG2* transgenes into the *VSG6* bloodstream expression site (BES) and consequent effect on growth of transgene containing cell lines. A. Site of integration of transgenes. Electroporation of Acc65I and SacI digested targeting construct (p3952; Supp. Fig. 1) containing either the wild-type or a mutant VSG2 ORF into bloodstream form trypanosomes expressing VSG6 and selection with blasticidin resulted in stable cell lines expressing either wild-type or mutant VSG2 in addition to wild-type VSG6. Sequence of the BES3 containing VSG6 is from Genbank FM162569; intact ORFs are indicated and the vertical ticks are spaced at 10 kbp. B. Map of VSG2 showing the location of PTCs, at codons 64, 167, 262, 392, 426 and 460. The signal peptide, the N-terminal domain, linkers (1 and 2), the C-terminal domain, and the GPI-anchor signal sequence, and the two N-linked oligosaccharides (NLO and residue number) are shown. The approximate molecular weights (kDa) of wild-type or each of the PTC-containing VSG monomers are shown.

Expression of a VSG protein is essential for the proliferation of bloodstream form trypanosomes [9]. To investigate any cytoplasmic control of *VSG* mRNA levels independently of cell viability, trypanosomes were made to express two *VSG* mRNAs from a single bloodstream form expression site (BES), generating double expressers, using a similar construct design to others [32] (Fig. 1A). This approach allowed the manipulation of one *VSG* mRNA whilst maintaining viability through expression of the endogenous VSG. In the experiments here, the parental cell line was *Trypanosoma brucei* Lister 427 expressing *VSG6* and the transgene encoded *VSG2* including the native 5′ and 3′UTRs (Supplementary Fig. 1).

In addition to a construct designed to express wildtype VSG2 after insertion into the active VSG6 expression site, a series of VSG2 mutants were made by introducing premature termination codons (PTCs) (Fig. 1B). VSG2 is encoded by a 476 codon open reading frame (ORF) and the mature VSG contains 433 amino acids after processing which removes a 26 residue N-terminal signal sequence and a 17 residue C-terminal GPI-anchor addition sequence [33]. Mature VSG2 has two folded domains, the N-terminal domain (residues 27–377, all numbering from the nascent polypeptide), an unstructured linker (378–400), the C-terminal domain (401–442) and a second linker (443–459) with the GPI-anchor attached. The PTCs in the VSG2 open reading frame were located at codons 64, 167 and 262 in the N-terminal domain, 392 in the interdomain linker, 426 in the C-terminal domain and 460, the first residue of the GPI-addition sequence designed to produce a mature VSG without a GPI-anchor (Fig. 1B).

**Fig. 2:**
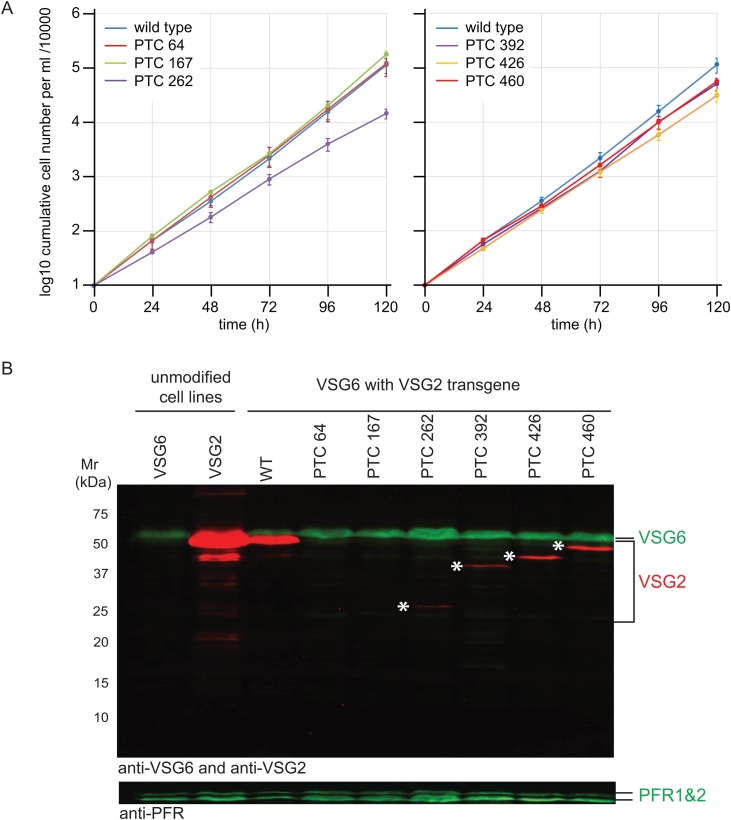
*VSG2* transgenes were expressed and PTCs were effective in terminating translation. A. Measurement of cell number over a five day time course for three independent clones of each transgenic cell line. Proliferation is expressed as cumulative cell number/10,000 and the standard error is shown (Supp. Table 2). Two panels are used for clarity. B. Western blot of whole cell lysates for the series of cell lines expressing a *VSG2* transgene in a VSG6 background. One clone of each cell line is shown: VSG6, the parental cell line expressing VSG6. VSG2, a distinct cell line expressing VSG2 from its endogenous locus. The remaining cell lines express a *VSG2* transgene in a VSG6 background: VSG6 VSG2 WT, or WT VSG6 with PTCs at one of the following codons within VSG2: 64, 167, 262, 392, 426 or 460 were loaded on the gel. Whole cell lysates of 2 × 10^6^ cell equivalents were loaded per lane and the white asterix (*) indicates truncated VSG2 polypeptides of the expected predicted molecular weights.

Three independent clones of each transgenic cell line were selected and any effect on proliferation was measured in continuous culture with daily passage over 5 days (Fig. 2A and Supp. Table 2). On averaging the three clones for any one cell line we observed that: (i) expression of a wild type *VSG2* transgene had little effect on proliferation when compared to the VSG6 expressing parental cell line, (ii) cell lines that contained the transgene with a PTC at codon 262 grew more slowly with a ∼10-fold reduction in population growth after 5 days as measured by cumulative cell number, (iii) the three cell lines with PTCs closest to the wild type stop codon (codons 392, 426 and 460) all had slightly reduced proliferation over 5 days, with 2- to 3-fold reduction in population growth as measured by cumulative cell number, (iv) PTCs at other locations had no effect.

Expression of VSGs in the cell lines was tested by western blotting for both the endogenous VSG6 and any product from the *VSG2* transgene (Fig. 2B). VSG2 was detected using an antiserum raised against a peptide corresponding to the N-terminal 15 residues of mature VSG2 [27], these residues were present in all VSG2s encoded by the PTC-containing transgenes. VSG2 polypeptides of the expected molecular weight were detected in cell lines containing PTCs at codons 262, 392, 426 and 460 as well as more abundant expression of the wild type *VSG2* transgene. No polypeptides were detected from *VSG2* transgenes with PTCs at 67 and 167 (Fig. 2B), these were assumed to be below the limit of detection in this experiment. These findings indicated that the *VSG2* transgenes were expressed as expected and that the PTCs were effective in terminating translation.

### 3.2 PTC-containing *VSG2* transgenes are subject to NMD and an additional pathway that detects non-functional VSG resulting in increased VSG mRNA

**Fig. 3:**
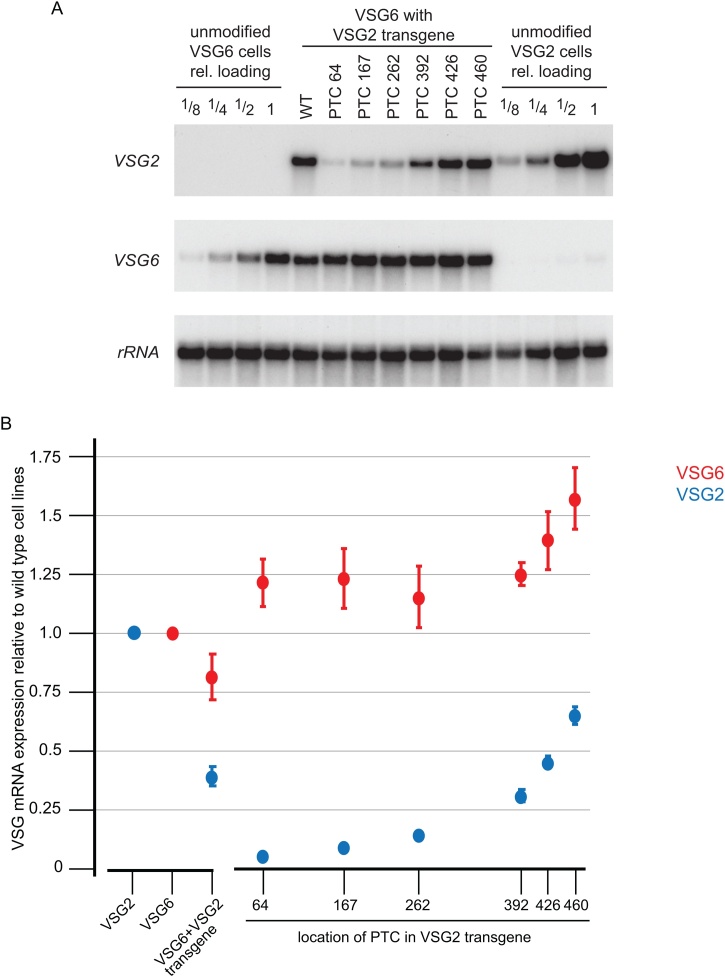
Expression of PTC-containing *VSG2* transgenes and measurement of transgene and endogenous *VSG* mRNA levels. A. Northern blot analysis of *VSG2* and *VSG6* mRNA levels in one set of cell lines expressing a *VSG2* transgene in a VSG6 background. To assist with quantitation, mRNA from unmanipulated VSG6 and VSG2 expressing cell lines was also analysed as a titration of the amount of total RNA loaded. A relative loading of 1 indicated 1 μg total RNA. The blot was probed for rRNA to adjust quantitation for precise loading. B. *VSG* mRNA levels determined from quantitative northern blots as above, expressed relative to the *VSG* mRNA level measured in unmodified cell lines VSG2 (blue) and VSG6 (red). First, an average value for cell lines expressing a wild type *VSG2* transgene in a VSG6 background (VSG6 + VSG2 transgene) is shown followed by a plot of *VSG* mRNA expression against the location of the stop codon in the *VSG2* transgene. The error bars are standard error of the mean from measurements of three independent clones for each transgenic cell line. The measurements of *VSG6* mRNA in all cell lines with a PTC-containing *VSG2* transgene were significantly different (p < 0.05 by Student’s unpaired t-test) to *VSG6* mRNA in cell lines with a wild type *VSG2* transgene (Supp. Table 3).

The expression of *VSG* mRNAs in cell lines containing each transgene was measured using quantitative northern blotting (Fig. 3A). mRNA levels were estimated as the average of three independent clones and expressed relative to the *VSG* mRNA from wild type cell lines expressing either *VSG2* or *VSG6* (Supp. Table 3), for example a value of 0.60 represents 60 % of the *VSG* mRNA present in the wild type cell line expressing that specific VSG. The values were then plotted against the location of the stop codon in the *VSG2* transgene (Fig. 3B).

The first observation from these measurements was that the insertion of the wild type *VSG2* transgene resulted in the expression of two VSG mRNAs as expected, but the levels of the *VSG2* and *VSG6* mRNAs were not the same, *VSG2* mRNA expression was 0.39 and *VSG6* mRNA was 0.81.

This observation was investigated further by using RNAseq to estimate *VSG* mRNA abundance as transcripts per million transcripts (TPM). RNA from wild type cell lines expressing either VSG2 or VSG6 was used along with a cell line expressing wild type VSG2 in a VSG6 background at two time points after electroporation of the transgene construct. RNAseq data was processed to produce estimates of mRNA abundance as transcripts per million transcripts (Table 1). In the wild type cell lines, *VSG* mRNA levels varied: *VSG2* mRNA represented 15 % and *VSG6* mRNA 22 % of total mRNA. In the cell line expressing transgenic VSG2, total *VSG* mRNA was intermediate with 18 % and 20 % of total mRNA respectively, and the ratio of *VSG* mRNAs was 0.26:0.74 (VSG2:VSG6) (Table 2).

**Table 1:** **Measurement of VSG mRNA abundance by RNAseq.** Two moderately abundant mRNAs (DHH1 and NOT1) an an abundant mRNA (RPL4) are shown for comparison.

mRNA expression in transcripts per million transcripts (TPM)					
Gene	Accession number	*T. brucei* L427 VSG2	*T. brucei* L427 VSG6 parental	*T. brucei* L427 VSG6:VSG2 d28	*T. brucei* L427 *VSG6:VSG2* d44
VSG6	Tb427.BES15.12	25	219777	136573	147716
VSG2	Tb427.BES40.22	151633	5	47619	51168
total VSG		151658	219782	184192	198884

DHH1	Tb427.10.3990	294	259	292	306
NOT1	Tb427.10.1510	197	161	135	155
RPL4	Tb427.03.5050	2214	1891	2202	2060

**Table 2:** The relative abundance of VSG mRNA in a transgenic cell line expressing wild type VSG2 compared to wild type cell lines.

Fractional VSG mRNA expression					
Gene	Accession number	*T. brucei* Lister 427 VSG6 parental	*T. brucei* Lister 427 VSG2	*T. brucei* Lister 427 VSG6 p3952 VSG6:VSG2 double expresser d28	*T. brucei* Lister 427 VSG6 p3952 VSG6:VSG2 double expresser d44
VSG6	Tb427.BES15.12	1	0	0.74	0.74
VSG2	Tb427.BES40.22	0	1	0.26	0.26

The RNAseq estimates of the abundance of *VSG2* and *VSG6* mRNAs were in agreement with the northern blot data. When the northern blot measurements were converted to abundance estimates, the ratio of mRNAs was 0.25:0.75, similar to that estimated by RNAseq (calculation in Supp. Fig. 2). Thus, the relative abundance of *VSG* mRNA can vary with the identity of the VSG and the decrease in *VSG6* mRNA on introduction of a *VSG2* transgene indicated that there is a regulatory system able to adjust the total *VSG* mRNA concentration. The adjustment could be occurring at the transcriptional and/or post-transcriptional level.

Next, the expression of *VSG2* mRNAs in cell lines with PTC-containing transgenes was measured by northern blotting (Fig. 3 and Supp. Table 3). The expression showed a position-dependent effect, with PTCs at codons 64, 167, 262, 392, 426 and 460 resulting in relative levels of 0.05, 0.09, 0.14, 0.31, 0.45 and 0.65 (Fig. 3B). The measurements of *VSG2* mRNA for the first four PTCs was as expected for an NMD pathway and provides further evidence that such a pathway is present in trypanosomes. However, an unexpected observation was that in the cell lines with PTCs at codons 426 and 460, the level of *VSG2* mRNA, 0.45 and 0.65 respectively, was greater than the wild type *VSG2* transgene, 0.39.

When expression of the endogenous *VSG6* mRNA was measured, two observations were made: first, expression of all the PTC-containing *VSG2* transgenes resulted in an increase in VSG6 mRNA from 0.81 for the wild type to at least 1.15, more than the amount present in the parental cell line (Fig. 3B). Second, for *VSG2* transgenes with PTCs close to the C-terminus, there was a larger increase to 1.39 and 1.57 for PTCs at 426 and 460 respectively (Fig. 3B). Thus, synthesis of a faulty VSG protein triggers a general increase in *VSG* mRNA.

## 4 Discussion

Trypanosomes tolerate the expression of two VSGs with little effect on growth in culture [32] and, as VSG expression is essential, investigations have used ectopic expression of a second VSG to investigate regulation. Here, transgenes encoding *VSG* mRNAs containing PTCs were used to trigger *VSG* mRNA decay in the cytoplasm and the effects on both transgene and endogenous *VSG* mRNA were measured. The insertion of a *VSG2* transgene upstream of the active *VSG6* gene (Fig. 1A) followed the original approach to successfully force the simultaneous expression of two VSGs [32], and was used here to ensure that both transgenic and endogenous VSGs were co-transcribed. In previous work [32], three different VSG transgenes were inserted upstream of the endogenous *VSG2* gene and, in each case, high levels of expression of VSG2 and the transgenic VSG were detected by immunofluorescence and western blotting [32]. Here, a *VSG2* transgene was inserted upstream of the endogenous *VSG6* gene and measurements of wild type transgenic and endogenous VSG expression by western blotting were similar to earlier work (Fig. 2B).

When the *VSG* mRNA levels were measured, the findings were: first, wild type cells expressing either VSG2 or VSG6 contained different amounts of *VSG* mRNA as a fraction of total mRNA: *VSG2* mRNA was 15 %, and *VSG6* mRNA was 22 %. Cells expressing a wild type *VSG2* transgene contained intermediate levels of *VSG* mRNA (Fig. 3 and Table 1). These measurements could arise from a genuine difference in VSG mRNA levels and/or result from differences in all other mRNAs, that is the amount of VSG mRNA remains constant but all other mRNAs change. It is not possible to rule out the latter but the former is more likely given probable folding efficiency variation in different VSG proteins discussed below.

Second, cell lines expressing a wild type *VSG2* transgene in a *VSG6* background contained a decreased amount of *VSG6* mRNA. However, the cells did not contain equal numbers of the two *VSG* mRNAs, the ratio was approximately 1:3 (*VSG2*:*VSG6*) measured by both RNAseq and northern blotting (Fig. 3 and Table 1).

Third, there was the expected position dependent response of *VSG2* mRNA levels to the inclusion of a PTC, the nearer the PTC to the N-terminus the lower the steady state levels of *VSG2* mRNA (Fig. 3). However, reduction in *VSG2* mRNA only occurred with PTCs located in the N-terminal domain or the inter-domain linker. This decrease in *VSG2* mRNA resulted in an increase in *VSG6* mRNA.

Fourth, *VSG2* transgenes containing a PTC in the C-terminal domain or at codon 460, which directed synthesis VSG2 without a GPI-anchor, resulted in both *VSG2* and *VSG6* mRNAs increasing 1.6 to 2-fold compared to the levels in cells expressing a wild type VSG2 transgene (Fig. 3). These variations in copy number indicate that it is unlikely that there is a direct counting mechanism for *VSG* mRNA, but rather a feedback from the production of functional VSG protein.

The first two findings concern the difference in *VSG* mRNA copy number in cells expressing different VSGs. Since the variation is present in the cell line expressing two VSGs from the same BES, it does not result from differential transcription and must be explained by post-transcriptional process(es). The major determinant of stability for most mRNAs in trypanosomes is codon use, expressed numerically as a gene expression codon adaptation index (geCAI) [26]. In this case, the geCAI value for *VSG2* is 0.33 and *VSG6* is 0.35, and this difference might contribute towards the higher levels of *VSG6* mRNA but is unlikely to be the sole determinant. The second observation that expression of a second VSG results in a reduction of the endogenous *VSG* mRNA has been reported before [16,17].

The expression of VSG2 with a PTC in the N-terminal domain or inter-domain linker resulted in the reduction in *VSG2* mRNA levels, inversely proportional to the length of the residual open reading frame (Fig. 3). This is a typical characteristic of an NMD pathway [23] and has been reported before in trypanosomes [24]. The decrease in *VSG2* mRNA resulted in an increase in *VSG6* mRNA to ∼1.5 fold more than cells expressing a full length *VSG2* transgene and ∼1.2 fold more than parental cells expressing VSG6. This increase to levels above that present in wild type cells indicated that the trypanosome has spare capacity for the production of *VSG* mRNA and is emphasised in cells expressing *VSG2* transgenes with PTCs in the C-terminal domain or immediately after the mature C-terminus, so that a complete but not GPI-anchored VSG was synthesised. In the latter case, both *VSG2* and *VSG6* mRNAs increased 1.6 to 2-fold.

One model arising from the measurements above is that *VSG* mRNA was subject to two pathways. First, an NMD pathway that decreased the amount of PTC-containing mRNA but became less effective when the PTC was close to the C-terminus. Second, a pathway that detected unsuccessful production of membrane-anchored VSG and triggered an increase in total *VSG* mRNA. The second pathway is apparent as an increase in *VSG6* mRNA in cells expressing VSG2 with an early PTC and an increase in both *VSG* mRNAs in cell expressing VSG2 with a late PTC, when the NMD pathway is less potent.

The kinetics and route of VSG synthesis are well characterised (reviewed in [34,35]). As VSGs are translated by ER-associated ribosomes, the PTC-containing *VSG* mRNAs would have directed translocation of a nascent VSG into the ER. It is likely that several could not fold correctly and were potential substrates for an unfolded protein response (UPR) pathway. However, there is evidence that the UPR is absent in bloodstream form trypanosomes [36] and that GPI-anchorless VSG is selectively retained and degraded in the lysosome [37,38]. Our favoured model for the function of the pathway that increases *VSG* mRNA in response to incorrect VSG protein is that it evolved to compensate for variation in the rates of successful folding of different VSGs. If only a small fraction of VSG protein folds successfully, the pathway increases *VSG* mRNA and if a larger fraction of the VSG folds correctly, then less *VSG* mRNA is produced. This model predicts that VSG2 folds more efficiently than VSG6. The model is also consistent with the observation that bloodstream form trypanosomes seem to have a capacity for degrading a large amount of non-functional VSG without any great effect on proliferation.

As *VSG* mRNAs are highly variable in sequence, how is the increased mRNA that occurred in response to the anchorless VSG specific to *VSG* mRNA? Different *VSG* mRNAs have one specific conserved sequence feature, the ‘16-mer’ element in the 3′ UTR [16] and this is an obvious candidate for mediating a specific response but the experiments here do not distinguish whether this happens in the cytoplasm via mRNA half-life and/or in the nucleus via transcription rate or more efficient mRNA maturation.

The strict selection pressure imposed by the mammalian immune response on bloodstream trypanosomes makes it likely that VSG synthesis is tightly regulated. Here, evidence is provided for sensors that detect the production of functional VSG coated membranes and a response that alters *VSG* mRNA levels. The regulation of VSG synthesis is central to the survival of the African trypanosome in a mammalian host and as such its regulation will contain further complexities.

## Author contributions

I.E.M. generated cell lines, performed proliferation experiments, performed sample preparation and subsequent western and northern blotting and analyses. S.K. performed RNA sequencing data analysis. A.S generated cell lines and prepared RNA for RNAseq. M.C. and A.S. devised and supervised the study. M.C. analysed data and wrote the manuscript. The final version of the manuscript was approved by all authors.
