# Supplementary material for: VSG mRNA levels are regulated by the production of functional VSG protein
Source: Mol Biochem Parasitol. 2021 Jan;241:111348. doi: 10.1016/j.molbiopara.2020.111348 (PMC7871013; doi:10.1016/j.molbiopara.2020.111348)
Supplement: Supplementary file 1 [file mmc1.docx]

Supplementary Figure 1A. Components in the insert of p3952, details are in the main text.


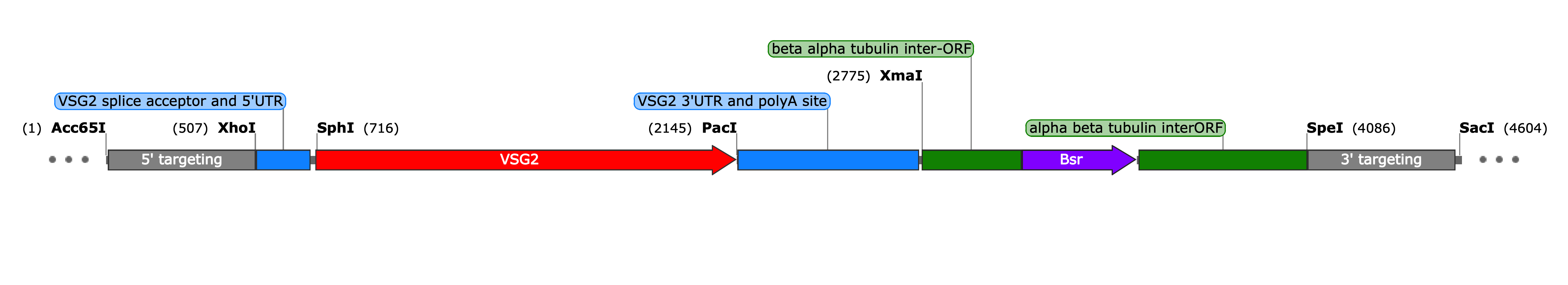


Supplementary Figure 1B

Sequence of insert from p3952

LOCUS Exported 4605 bp ds-DNA

DEFINITION .

ACCESSION .

VERSION .

KEYWORDS .

SOURCE synthetic DNA construct

ORGANISM synthetic DNA construct

REFERENCE 1 (bases 1 to 4605)

AUTHORS .

TITLE Direct Submission

JOURNAL Exported Thursday, Apr 23, 2020 from SnapGene 5.1.0

https://www.snapgene.com

FEATURES Location/Qualifiers

source 1..4605

/organism="synthetic DNA construct"

/mol_type="other DNA"

ORIGIN

1 ggtaccct gttctaaaca taaacatagc aataaatatt atcggaaacg agcaaataca

61 ggaaggttgt aattgaaata gagtcactaa attatccggt aaaccagtgg aagtaatagt

121 aaaattaaca aaagtataga atgaaataat gtgactgtca acaacttccg cagaacaagg

181 gaaaataata catgaagagg aagaggagat aatgcaccgt atttcactca gaaatggata

241 tattattttg ccgtggtggt ggcgtctatg tttcgttggc tgatgacgga ttctaatgtt

301 cagttgttga caataagcaa ctccaaagca cacaatggta aacaaaggaa atggaacaaa

361 aggaaagtgc ttgaaaagat tgcagtccta caacaaaata caaaaattgc agtaagaaaa

421 cacagagcag ctgtgatcac tgccttgact gaggatatat ttttcggaat cgaaaaacta

481 atcgtttata aaattagaaa agcactgact cgagcacaca agcattctat acgtaaaaga

541 tctagtatat aggagcaacg ctctgccaaa acataatggc aagacaaacg gccgtgtttg

601 ccgctgatgc tacagaacca gcttaatttc cagaagacga aaatttgcat gttttcccac

661 aatattttaa ttactcttga agattgtagt tattcctacg cgacacgaac gcggcatgcc

721 ttccaatcag gaggcccggc ttttcctcgc cgtcttggtc ctagcccaag ttcttccaat

781 tcttgtcgat tcggcggctg aaaaaggttt caaacaagct ttttggcaac ctctttgcca

841 ggtctccgag gagctagacg accaaccgaa gggtgcgttg tttacgctgc aagcagcggc

901 gagcaaaatc cagaaaatga gggacgcggc actgcgagca agtatatacg ctgaaataaa

961 tcacggcacc aacagggcca aggcagccgt tatagtcgcc aaccactatg ccatgaaagc

1021 tgatagcggc ctagaggccc taaaacaaac gttaagcagc caagaggtaa cagctactgc

1081 aacagcgagc tacctaaaag gaagaataga cgaataccta aatctccttc tacaaacaaa

1141 ggagagcggc accagcggct gcatgatgga caccagcgga acaaacacgg taacgaaggc

1201 cggcggcacc atcggaggcg ttccttgcaa gctgcagttg tcgccgatac agccgaagcg

1261 acccgcagcg acctacctag gtaaagcggg ctacgtaggc ctaacacgac aagcagatgc

1321 agccaacaat ttccacgata acgacgccga atgcaggcta gccagtgggc acaacaccaa

1381 cggcctcggc aaaagcggcc agctttctgc agcggtcact atggcggccg gctatgtcac

1441 agtagcgaac agccaaacag ccgtcacggt ccaggcgctc gatgcattac aggaagcgag

1501 cggagcagcg caccaaccgt ggatcgacgc ctggaaggcc aagaaagcgc taacaggagc

1561 agaaaccgct gagttcagaa acgaaacagc cggaatagct ggcaaaacag gcgttaccaa

1621 gcttgttgaa gaagctttac taaagaaaaa agactcagag gcctcagaaa tacaaacaga

1681 attaaaaaaa tactttagcg gccacgaaaa tgaacagtgg acagcaatag aaaagctcat

1741 atccgagcag ccagtggcgc aaaacctggt aggcgacaac cagccaacca agctagggga

1801 actggagggc aatgccaagt taacgactat acttgcctat taccgaatgg aaacagcagg

1861 gaaatttgaa gttttaaccc agaagcacaa gcccgctgaa agccaacaac aagcagcaga

1921 aacagaaggc agctgcaaca agaaggacca aaatgagtgc aaatccccat gcaaatggca

1981 taacgatgcg gaaaacaaaa agtgcacatt ggataaggag gaggcaaaaa aggtagcaga

2041 tgagactgca aaagatggga aaactggaaa cacaaacacc acaggaagca gcaattcttt

2101 tgtcattagc aagacccctc tttggcttgc agttttgctt ttttaattaa tttcccccct

2161 caaatttccc ccctcctttt aaaattttcc ttgctacttg aaaacttttt gatatatttt

2221 aacaccaaaa ccagccgaga ttttgtgttc tgtgttttgt aagttgactg tctgattgtc

2281 tagaaatatt ttctggcaac taaaattttt ttcttttttc ctgttttttt tgtaggtagg

2341 taggaatggg ggggggggta gttaggtagg ttagttaggt tagttagggg gttagttagg

2401 ggggttaggc ttaggattag gattagactt aggcttagga ttaggattag gattaggatt

2461 agggttaatt ttttcctctt tttttttaac tcacacctct atcctggatt tttaattttt

2521 ttttttagcc attcgcggct cctttttttt tttttgcgcc aatgtttaat tttttattgt

2581 gttttcaatt tttttgtcaa ccatgcagcg gctgttttgt tatgcggacc ctaaccctcc

2641 tccccccccc ccgcccgcgc acctccattt ttaaaaattt ttttaccgcg tccttcaacc

2701 agaatttttt taaatttttt aatttttttt attttccgtg gttttgaatc ttaatttttc

2761 gggggaattc ctgcagcccg ggtagaaagt gtgacaacgt cgcaccatgt gtaggttttc

2821 atttatgttc tttctttctt ttttttgtga atttgttttc tgtctcaaat gtttttaatt

2881 cgcttgggac ctatgttttt cttgtttttt tgctcaccct ttgtgtagga ggcaccctgt

2941 cacgtctgtg gttgcgtgta tgccttcctt ccccttattc gcttcttcct gtcgtgtcac

3001 acctctttct ctctctccct ttccgccttt tctttcaatc ttgttttctc gaccagccct

3061 actagaggag aaagaacagt aaccctttca tcaaagaaaa tagttcaaac gaattcatat

3121 gcctttgtct caagaagaat ccaccctcat tgaaagagca acggctacaa tcaacagcat

3181 ccccatctct gaagactaca gcgtcgccag cgcagctctc tctagcgacg gccgcatctt

3241 cactggtgtc aatgtatatc attttactgg gggaccttgt gcagaactcg tggtgctggg

3301 cactgctgct gctgcggcag ctggcaacct gacttgtatc gtcgcgatcg gaaatgagaa

3361 caggggcatc ttgagcccct gcggacggtg ccgacaggtg cttctcgatc tgcatcctgg

3421 gatcaaggcc atagtgaagg acagtgatgg acagccgacg gcagttggga ttcgtgaatt

3481 gctgccctct ggttatgtgt gggagggcta attcgaatag acgcggacgg ggcatttccc

3541 gttcgtcatt agcagtaggt aatgaagatg tttgtttctc gtcccctttc tccttcgtcc

3601 ttctgtcatt ttgttctttt gtgtttatgt tttgttgttg ttttctttaa tttttttttt

3661 tcttccacgt ttgtgtacat ccgcgcgcca ctctattcag agagccacgg atagtagagg

3721 aggtgggaag ggtatatgag ggacacgcgt accatgatgt gggatgtatt ggggtccctg

3781 tctgtcctta cgtgactatg tatgaaccgt cacgtgtaag atgagctagt gagatcaaca

3841 gtacaactca tcaacacgcc ttcttctcgt taaatgtaca caatcttgat cctccacctt

3901 tatgggtccc attgtttgcc tcttccgctg tgtggagtgc gcctacacgc acttctcact

3961 tcgtaagtgg tggtggcgta agtattgcct aatgttgact ctatattctc ctctcctcac

4021 cccctcgcgg tgctgatttc tgacagatct tcaaacacta gattaagcaa aggactattc

4081 atccgttact agtaccacct gtgcgacgaa gctgcaaaag aataaagcgg caccaagtag

4141 ttctaacaag ctgtagtgag acccagctgc acgatagatt aatttattat tttttaaatt

4201 ttgagttttt ttaaatttat ataagtgtaa ttcaccacat taaaaagggg gaaagaggac

4261 tcaaaatgat attctaatta gctgaaggaa acggtgatga aaatagataa taaacattcc

4321 ccaaaaatat taccacaact aacttctttg tttttctctt tcatgttgct aaactagaca

4381 acagcgttaa gcgatggccg tgcacagagc cctagcggcg tacgcgatta gtctttacgt

4441 tttactaccc agaaaatcgg gagcaacaga caaaggcgcg atcaagtttg agacgtggga

4501 gccgctctgt ttactgacac aagacttcgg taacctttac aacagagcgc acaaacttaa

4561 tctcgacatc gacacctacg taaccgcagc ccaccgcggt ggagc

//

Supplementary Figure 2

Conversion of northern blot VSG mRNA measurements in cells expressing a wild type VSG2 transgene in a VSG6 background to mRNA molecules in TPMs using RNAseq data

TPM = TPM in unmodified cell lines x relative expression in transgenic cell lines

measured by northern blot

Copy number (TPM) of VSG6 mRNA = 220 000 x 0.81 = 178 000

Copy number (TPM) of VSG2 mRNA = 150 000 x 0.39 = 59 000

**Fraction of total VSG mRNA**

VSG2 = 59/(178 + 59) = 0.25

VSG6 = 178/(178 + 59) = 0.75
